# Reduction of extrinsic tooth stain by a toothpaste containing 10% high cleaning silica, 0.5% sodium phytate and 0.5% sodium pyrophosphate: an 8-week randomised clinical trial

**DOI:** 10.1186/s12903-021-01484-5

**Published:** 2021-03-11

**Authors:** Hongchun Liu, Jiazhen Tu

**Affiliations:** 1 Department of Preventive Dentistry, Hospital of Stomatology, Guanghua School of Stomatology, Sun Yat-sen University, 56 Ling Yuan Road W., Guangzhou, 510055 China; 2 Guangdong Provincial Key Laboratory of Stomatology, Sun Yat-sen University, Guangzhou, 510080 China

**Keywords:** Clinical trial, Toothpastes, Tooth discoloration, Phytate acid, Pyrophosphate

## Abstract

**Objective:**

To assess the effects for controlling extrinsic tooth stain of a whitening toothpaste containing 10% high cleaning silica, 0.5% sodium phytate and 0.5% sodium pyrophosphate, in comparison with a negative control toothpaste.

**Methods:**

A total of 86 adults who met with the inclusion and exclusion criteria were invited to take part in the study. They were distributed into test and control groups randomly. At baseline, 4 weeks and 8 weeks, the same examiner provided the clinical examinations, including evaluations of oral soft and hard tissues and measurements of tooth stain of the anterior teeth using the Lobene Stain Index. Adverse events and any changes in general health conditions of the patients were monitored.

**Results:**

When the study was completed, comparisons between patients in test and control groups yielded statistically significant differences in Lobene stain adjusted mean area score [0.83 (0.05) vs. 1.13 (0.05)], Lobene stain adjusted mean intensity score [0.99 (0.06) vs. 1.32 (0.06)] and Lobene stain adjusted mean composite score [1.45 (0.13) vs. 2.50 (0.13)] (All, *P* < 0.001). Patients in the test group exhibited reductions of 26.55%, 25% and 42%, respectively in Lobene stain area, intensity and composite scores, relative to patients in the control group. Comparisons within groups showed that all three Lobene scores at 8 weeks in both groups were lower than those at baseline (All, *P* < 0.001).

**Conclusion:**

This study demonstrates that 8-week use of a toothpaste containing 10% high cleaning silica, 0.5% sodium phytate and 0.5% sodium pyrophosphate can effectively reduce extrinsic tooth stain.

*Trial registration* NCT04238429 (before enrollment of the first participant). Data register: March 4, 2018.

## Introduction

Extrinsic tooth stain is a common esthetic problem, especially on anterior teeth. Epidemiological studies in different populations found that 17.9–52.6% of the subjects dissatisfied with their tooth color [1–4], adults in younger ages had greater dissatisfaction with dental appearance or color in comparison with adults over 55 years old [2]. Therefore, there is great desire for patients to achieve whiter teeth, which would provide a bright smile, increase personal attractiveness and improve quality of life [5].

Extrinsic tooth stain is caused by deposition of colored compound called chromophores [6, 7]. Chromophores can be absorbed directly on the enamel or exposed dentin surfaces [8], especially rough surfaces that have thick pellicle layers and are difficult to clean. They can also be incorporated into the acquired pellicle, biofilm or calculus. They fall into two categories: organic and inorganic. Organic chromophores are small organic molecules from tea, coffee, red wine or fruits [9]. They have conjugated bonds in their chemical structure and a high affinity to protein inside plaque and pellicle. Inorganic chromophores are colored metals, such as copper, nickel and iron [10]. The individuals who obtain these inorganic chromophores are workers in the copper, iron and nickel industries, those using mouth rinses containing copper salts and those taking iron supplements. Besides, various factors are in favor of stain formation. Inadequate toothbrushing is detrimental to oral hygiene and allows the accumulation of stained pellicle and chromogen deposits. However, oral care products such as stannous fluoride and chlorhexidine could cause indirect stain. There is a precipitation reaction between cationic chlorhexidine and dietary anionic chromogens from foods and drinks such as tea and coffee [10, 11]. Stannous fluoride in toothpaste may hydrolyze or oxidize because of its unstablized formulations, and promote a yellowish-brown stain [11, 12].

In recent years there is great interest on the treatment of tooth stain with various tooth whitening agents, among which toothpaste with active ingredients is widely used. Firstly, abrasive is formulated to remove dental plaque. It is the primary stain removal component in almost all toothpastes. Hard abrasives remove stains more efficiently than soft ones, however, they may be harmful to the enamel, exposed dentin and gingiva if they are too abrasive. Secondly, anti-redeposition agents such as polyphosphates prevent chromophore deposition and inhibit calculus formation where external stains could be incorporated [7, 13]. They have a strong adsorption affinity to the tooth surface, bind colored inorganic ions and prevent their deposition, and prevent the subsequent deposition of chromophores after the whitening procedure. Pyrophosphate is a linear condensed polyphosphate that is widely used in whitening toothpaste. Its chelating ability provides a strong binding affinity for calcium salts in tooth surface and dental calculus [14], which makes it desorb adsorbed pellicle proteins that trap stain. Pyrophosphate reduces formation of dental calculus that acquires tooth stains, as it block calcium sites for crystal growth by adsorbing onto the surfaces of hydroxyapatite crystal [15]. Sodium phytate is a cyclic organic hexa-phosphate and the principal storage form of phosphorous molecule in plant seeds [16, 17]. It shares similar chemical characteristics with pyrophosphate as it has multiple phosphate groups. Toothpaste and mouthwash containing phytate are found to reduce tooth stain accumulation [17] and dental calculus formation [18].

This 8-week randomised clinical trial was aimed to assess the effects for controlling extrinsic tooth stain of a whitening toothpaste containing 10% high cleaning silica, 0.5% sodium phytate and 0.5% sodium pyrophosphate, in comparison with a negative control toothpaste containing conventional silica base without any active ingredient.

## Materials and methods

### Study population and design

This clinical trial was conducted at Guanghua School of Stomatology, Sun Yat-sen University, Guangzhou, China, from May to October 2018. The study protocols were approved by the institutional review board at Guanghua School of Stomatology, Sun Yat-sen University and were in good accordance with the World Medical Association Declaration of Helsinki on ethical aspects. This eight week study used a randomised, double-blind, parallel-group design. It was registered at ClinicalTrials.gov as NCT04238429 first on March 4, 2018.

According to the data from a previous clinical study on tooth stain removal efficacy of a whitening toothpaste [19], the sample size of this study was calculated. Based on a standard deviation of 0.5 for Lobene stain area score, a reduction in the Lobene stain mean area scores of 20% between the test and control groups, a power of 80%, a significance of 0.05, and an attrition of 10%, it was estimated that 40 patients needed to be included in each group.

The participants were informed of the study purposes and told that their participation was voluntary. Informed consent forms were signed. A total of 86 adults who met with the inclusion and exclusion criteria were invited to take part in the study. The inclusion criteria were: (1) aged 18–70 years old; (2) good general health; (3) able to attend the clinical examinations during the eight week study period; (4) possessing more than 20 natural permanent teeth that were uncrowned or not heavily filled with restorative materials, excluding third molars; (5) having at least 12 anterior teeth that are suitable for scoring (without crowns, veneers or large restorations, not traumatic teeth or unvital pulp teeth); (6) having a whole-mouth mean tooth stain composite score of more than 1.5 according to the Lobene Stain Index [20]. Exclusion criteria were: (1) severe oral diseases or chronic diseases; (2) advanced periodontal diseases; (3) pregnant or lactating females; (4) fluorosis or tetracycline teeth; (5) wearing orthodontic bands or partial or removable dentures; (6) taking part in other clinical trials; (7) receiving prophylaxis during the previous 3 months or tooth whitening treatment during the past 6 months; (8) allergic to the study products.

### Study procedures

Eligible patients were given computer-generated random numbers in ascending numerical order as they were enrolled in the study by one external dentist. They were distributed into test and control groups randomly. This dentist kept the randomization and group allocation information. All the other study personnel did not have access to the information during the course of the study.

The patients in the test group were given a test toothpaste containing 10% high cleaning silica, 0.5% sodium phytate and 0.5% sodium pyrophosphate, while the patients in the control group were given a negative control toothpaste containing conventional silica base without any active ingredient. All toothpastes were identical in appearance and taste. The toothpaste was marked with its specific code number which represented its group allocation and this number was not decoded until the end of the study. The distribution of the toothpaste was made in a separate place to ensure that other study personnel and patients were unaware of group assignment.

All of the patients were provided with the same adult soft-bristled toothbrushes, in order to maintain uniformity between groups. The patients were asked to only use the assigned toothpaste and the same toothbrush to brush their teeth twice daily, in the morning and in the evening, for one minute. The patients were asked to maintain their other oral hygiene habits and daily eating habits. The patients were not allowed to use any other toothpastes and oral health care products, such as mouth rinse.

The patients came to the dental clinic to receive clinical examinations at baseline, 4 weeks and 8 weeks. The same examiner provided the clinical examinations during the course of the study. Firstly, evaluation of oral soft and hard tissues was performed. The examiner assessed soft and hard palate, oral mucosa, tongue, sublingual and mandibular areas, salivary glands and pharynx and larynx areas of each patient. Secondly, tooth stain of the anterior teeth were measured using the Lobene Stain Index [20]. The index measures the area and intensity of extrinsic tooth stain on the facial and lingual surfaces of six lower anterior teeth and facial surfaces of six upper anterior teeth. The tooth surfaces were divided into two regions: body and gingival. For each part, the intensity and extent of extrinsic tooth stain were graded on a scale of 0–3. For stain intensity, the scale is scored as follows: 0 = No stain; 1 = Light stain (yellow to light brown or gray); 2 = Moderate stain (medium brown); and 3 = Heavy stain (dark brown to black). For stain area, the scale is scored as follows: 0 = No stain detected; 1 = Stain covering up to 1/3 of the region; 2 = Stain covering > 1/3–2/3 of the region; and 3 = Stain covering > 2/3 of the region. Three variables on tooth stain were calculated for each patient. The Lobene stain mean area, intensity and composite scores were the sum of the scores on the recorded regions of tooth surfaces divided by the number of regions. The stain composite score for each region of the tooth surfaces was the stain area score multiplied by the stain intensity score.

During the follow-up examinations at 4 weeks and 8 weeks, the patients were also questioned by an investigator if they had experienced any adverse events. The adverse events included discomfort while brushing, bitter or alteration in taste, any allergic reactions arising from the use of the toothpastes and any feeling of changes on oral soft tissues. Any changes in general health conditions of the patients that were product related were also monitored.

After the patient finished each examination session, the acquired data were collected by the facilitator who kept the data. The other study personnel had no way to access the patient data during the course of the study.

The calibration of intra-examiner for the clinical examinations was made by repeating examinations on teeth in 10 patients before the study. There was perfect agreement in the classifications of Lobene stain area scores (the weighed kappa = 0.86) and Lobene stain intensity scores (the weighed kappa = 0.84).

### Statistical analysis

Chi-square test and the independent *t* test were conducted to compare the differences between treatment groups on sex, age and baseline Lobene stain scores. ANCOVA with baseline Lobene stain score as the covariate was conducted to compare the differences between groups in follow-up Lobene stain area, intensity and composite scores. For each group, paired *t* tests were conducted to compare the differences between baseline and each of the two follow-ups in Lobene stain scores. All statistical analyses were two sided and at a significance level of *a* = 0.05.

## Results

**Fig. 1:**
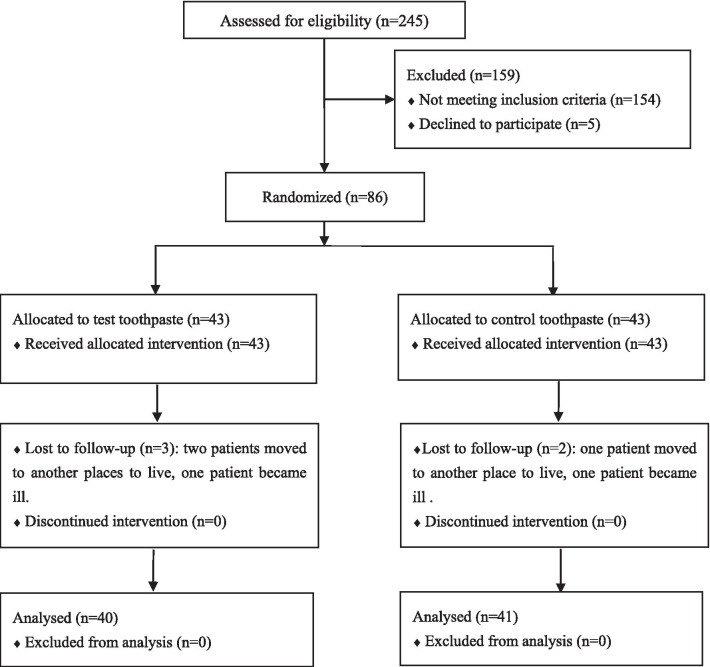
Flow of participants through each stage of the trial

A total of 86 patients took part in this trial, with 43 patients in each group. In the test group, two patients moved to another place to live and one patient became ill. They could not attend the follow-up examinations. In the control group, one patient moved to another place to live and the other became ill. The withdrawals did not relate to the use of products. 81 patients completed the 8-week clinical trial. Among them, 40 patients were in test group and 41 patients were in control group. Figure 1 depicts the detailed flowchart of the patients in the trial.

Demographic characteristics of the patients who completed the trial are described in Table 1. There were no statistically significant differences in age and gender between the test and control groups (*P* = 0.84 and *P* = 0.32, respectively), which demonstrated that proper randomization was conducted in this study.

**Table 1:** Demographic characteristics of the patients who completed the study

Groups	Gender		P*	Age (years)	P*
	Male	Female		Mean ± SD	
Test (n = 40)	23	17	0.32	42.50 ± 9.22	0.84
Control (n = 41)	19	22		42.05 ± 10.19	

*t test for comparing age between groups and Chi-square test for comparing gender between groups

*SD* standard deviation

Baseline Lobene stain scores of the patients who completed the trial were demonstrated in Table 2. No statistically significant differences in Lobene stain area, intensity and composite scores were found between test and control groups (*P* = 0.75, *P* = 0.81 and *P* = 0.96, respectively).

**Table 2:** Baseline Lobene stain scores of the patients who completed the study

Variables	Test toothpaste	Control toothpaste	P*
Lobene stain area score	1.63 (0.08)	1.59 (0.07)	0.75
Lobene stain intensity score	1.69 (0.10)	1.72 (0.09)	0.81
Lobene stain composite score	3.70 (0.26)	3.72 (0.24)	0.96

Data are mean (SE: standard error)

*t test

The changes of Lobene stain scores at 4 weeks of the patients who completed the trial were described in Table 3. Comparisons between test and control groups yielded statistically significant differences in Lobene stain adjusted mean area score [1.09 (0.05) vs. 1.33 (0.05), *P* = 0.002], Lobene stain adjusted mean intensity score [1.37 (0.06) vs. 1.63 (0.06), *P* = 0.004] and Lobene stain adjusted mean composite score [2.39 (0.16) vs. 3.23 (0.16), *P* < 0.001]. Patients in the test group exhibited reductions of 18.05%, 15.95% and 26.01%, respectively in Lobene stain area, intensity and composite scores, relative to patients in the control group. Comparisons within groups showed that Lobene stain area, intensity and composite scores at 4 weeks in the test group were lower than those at baseline (All, *P* < 0.001), and all three Lobene scores at 4 weeks in the control group were lower than those at baseline ( *P* < 0.001, *P* = 0.01 and *P* < 0.001).

**Table 3:** Summary of Lobene scores at 4 Weeks of the patients who completed the study

Variables	Test toothpaste	Control toothpaste	Difference between groups (%)	*P* between groups*
Lobene stain area score				
Adjusted mean (SE)	1.09 (0.05)	1.33 (0.05)	18.05%	0.002
95% Confidence interval	(0.98, 1.19)	(1.22, 1.43)		
Change from baseline (%)	33.13%	16.35%		
* P* versus baseline^†^	< 0.001	< 0.001		
Lobene stain intensity score				
Adjusted mean (SE)	1.37 (0.06)	1.63 (0.06)	15.95%	0.004
95% Confidence interval	(1.24, 1.49)	(1.50, 1.75)		
Change from baseline (%)	18.93%	5.23%		
* P* versus baseline^†^	< 0.001	0.01		
Lobene stain composite score				
Adjusted mean (SE)	2.39 (0.16)	3.23 (0.16)	26.01%	< 0.001
95% Confidence interval	(2.08, 2.71)	(2.92, 3.54)		
Change from baseline (%)	35.4%	13.17%		
* P* versus baseline^†^	< 0.001	< 0.001		

*ANCOVA

^†^Paired *t* test

The changes of Lobene stain scores at 8 weeks of the patients who completed the trial were described in Table 4. Comparisons between test and control groups yielded statistically significant differences in Lobene stain adjusted mean area score [0.83 (0.05) vs. 1.13 (0.05)], Lobene stain adjusted mean intensity score [0.99 (0.06) vs. 1.32 (0.06)] and Lobene stain adjusted mean composite score [1.45 (0.13) vs. 2.5 (0.13)] (All, *P* < 0.001). Patients in the test group exhibited reductions of 26.55%, 25% and 42%, respectively in Lobene stain area, intensity and composite scores, relative to patients in the control group. Comparisons within groups showed that all three Lobene scores at 8 weeks in both groups were lower than those at baseline (All, *P* < 0.001).

**Table 4:** Summary of Lobene scores at 8 Weeks of the patients who completed the study

Variables	Test toothpaste	Control toothpaste	Difference between groups (%)	*P* Between groups*
Lobene stain area score				
Adjusted mean (SE)	0.83 (0.05)	1.13 (0.05)	26.55%	< 0.001
95% Confidence interval	(0.72, 0.93)	(1.02, 1.23)		
Change from baseline (%)	49.08%	28.93%		
* P* versus baseline^†^	< 0.001	< 0.001		
Lobene stain intensity score				
Adjusted mean (SE)	0.99 (0.06)	1.32 (0.06)	25%	< 0.001
95% Confidence interval	(0.87, 1.12)	(1.20, 1.44)		
Change from baseline (%)	41.42%	23.26%		
* P* versus baseline^†^	< 0.001	< 0.001		
Lobene stain composite score				
Adjusted mean (SE)	1.45 (0.13)	2.50 (0.13)	42%	< 0.001
95% Confidence interval	(1.19, 1.72)	(2.24, 2.76)		
Change from baseline (%)	60.81%	32.8%		
* P* versus baseline^†^	< 0.001	< 0.001		

*ANCOVA

^†^Paired *t* test

No adverse events were reported by the patients or observed by the examiner throughout the study.

## Discussion

This study assesses the stain removal efficacy of a toothpaste containing 10% high cleaning silica, 0.5% sodium phytate and 0.5% sodium pyrophosphate. In our study we used the Lobene stain index that is based on a visual inspection of tooth color. After 4 weeks of twice-daily home use, there were statistically significant differences in the reduction of Lobene stain area, intensity and composite scores between test and control toothpastes (*P* = 0.002, *P* = 0.004, *P* < 0.001). The better effects on extrinsic tooth stain of the test toothpaste continued at 8 weeks. When the study was concluded, the Lobene stain area, intensity and composite scores in the test group improved 26.55%, 25% and 42%, respectively compared with those in the control group. These findings demonstrated that the toothpaste with the above formulation was efficacious in reducing extrinsic tooth stain after 8 weeks of home use. However, these results should be considered with caution because there is no active control in this study. Further study with active controls should be used.

In literature the efficacy of whitening toothpastes is controversial [21]. Some studies found that they are not effective in clinical trial [22] and in vitro study using bovine tooth discs [23]. Other clinical trials, including this study, found that whitening toothpastes had a beneficial effect in reducing extrinsic tooth discoloration [24]. A systematic review included eligible clinical trials of 32 comparisons between whitening dentifrice and regular dentifrice. It was found that the difference of mean stain area between two dentifrices was a reduction of − 0.44 according to the original Lobene Stain Index in favor of the whitening dentifrice [24].

Chromophores in tooth stains absorb light in the visible range and reflect mainly the complementary color that is recognized by the eyes, typically yellow or brownish in the case of teeth [7]. Abrasive is one of the most important ingredients in whitening toothpaste for mechanical removal of extrinsic stains. Abrasives include sodium bicarbonate, calcium carbonate, calcium pyrophosphate, hydroxyapatite, hydrated silica and etc. [5]. Through physical actions during brushing movement, they remove tooth stain. To achieve better effects, harder abrasives or higher amount of abrasives were added into the whitening toothpaste. However, high abrasivity would bring potential damage to and removing the outer layer of the tooth structure when brushing [7]. Formulation is a compromise between clinical cleaning efficiency and unwanted tooth abrasion. Therefore, in this toothpaste formulation, only a moderate amount of high cleaning silica is incorporated.

Incorporation of antideposition agents such as pyrophosphate and sodium phytate helps to improve whitening effects without increasing abrasive amount in the toothpaste. It also helps to clean those tooth areas that are hard to access and less effectively be cleaned by toothbrush bristles. There are very few studies in the literature that reported the stain removal efficacy of toothpaste containing sodium phytate. A previous study has found that the dentifrice with the sodium phytate formulation provides significant reductions of Lobene stain index (MacPherson modification) mean area and intensity scores compared with the reference dentifrice [17].

This study showed some effects of the negative toothpaste. One potential reason is that if both examiners and participants believe a whitening toothpaste was used (whether they were in the control group or test group), they may alter their scores resulting in a reduction also observed in the control group. Another possible reason maybe the Hawthorne effect. Frequent oral examinations during the course of study may enhance patients’ awareness of good oral hygiene practices, which helps to reduce extrinsic tooth stain.

In this study there were 3 active ingredients in the toothpaste formulation. Because of funding limitations, we did not include control groups that using single additive toothpaste, such as 10% high cleaning silica alone, 0.5% sodium phytate alone and 0.5% sodium pyrophosphate alone. It is not possible to distinguish which active ingredient is responsible for the outcome and to what extent, or if the combination was more or less efficacious than the single additive toothpaste. In the future research, we hope to include more control groups to address this problem.

## Conclusions

This study demonstrates that 8-week use of a toothpaste containing 10% high cleaning silica, 0.5% sodium phytate and 0.5% sodium pyrophosphate can effectively reduce extrinsic tooth stain compared to a negative control toothpaste. Additional studies with similar design are needed in which observation time is prolonged or various control toothpastes are included to observe the possible longer-term effects and relative effectiveness.

## Data Availability

The datasets used and/or analyzed during the current study are available from the corresponding author on reasonable request.
